# Knowledge, Beliefs and Attitudes towards the Influenza Vaccine among Future Healthcare Workers in Poland

**DOI:** 10.3390/ijerph18042105

**Published:** 2021-02-22

**Authors:** Sylwia Kałucka, Agnieszka Głowacka, Elżbieta Dziankowska-Zaborszczyk, Izabela Grzegorczyk-Karolak

**Affiliations:** 1 Department of Coordinated Care, Medical University of Lodz, 90-251 Lodz, Poland; 2 Department of Developmental Nursing and Health Promotion, Medical University of Lodz, 90-251 Lodz, Poland; agnieszka.glowacka@umed.lodz.pl; 3 Department of Epidemiology and Biostatistics, Medical University of Lodz, 90-647 Lodz, Poland; elzbieta.dziankowska-zaborszczyk@umed.lodz.pl; 4 Department of Biology and Pharmaceutical Botany, Medical University of Lodz, 90-151 Lodz, Poland; izabela.grzegorczyk@umed.lodz.pl

**Keywords:** flu vaccine, knowledge, beliefs, attitudes, nursing, public health, pharmacy, midwifery, students, survey

## Abstract

The flu vaccine is the best treatment for avoiding the flu and its complications. The aim of the study was to evaluate the knowledge of the flu vaccine and attitude towards the influenza vaccine among medical students in four majors of study (Nursing, Midwifery, Pharmacy, and Public health) in all years of study. A total number of 1137 subjects took part in the study. Most of the vaccinated students assessed the flu vaccine positively (78.5%, 73.7%, 60.7%, and 65.1%, according to their respective majors) and reported that they did not get the flu during the period of vaccination (90.4%, 92.1%, 87.4%, and 97.7%, respectively). Therefore, 65% of the students of Pharmacy, 78% of Midwifery, and 83% of Nursing who were vaccinated once in the last three years recommended the influenza vaccination, and 100% of all students received a regular vaccination every year. The univariate and multivariate logistic regressions showed that a maximum of four factors had a significant impact on the students’ knowledge of the influenza vaccine. Knowledge about the flu vaccine was the highest among Pharmacy students and lowest among Public health students. Final-year students answered the questions better than the younger ones (*p* < 0.05). Their place of residence and flu vaccination status also appeared to influence their answers. Although all students demonstrated good knowledge of the flu vaccine and demonstrated positive attitudes towards the vaccine, their rate of immunization was low. Therefore, health promotion programs are needed to improve immunization coverage among medical students who are future healthcare workers.

## 1. Introduction

The flu vaccine is the best remedy for reducing the burden of influenza for individuals and society. Healthcare workers (HCWs) who vaccinate themselves against influenza protect not only themselves but, also, patients against flu (as high-risk groups). Therefore, they prevent the spread of the flu and promote positive health behaviors in society [1]. The vaccination in this group is an important predictor of uptake in society. It is more important that, despite the widespread availability of the vaccine and the annual returning of the flu epidemics, a low coverage of vaccination has still remained in many countries. It is estimated that 5%–10% of adults and between 20%–30% of children suffer from influenza every year worldwide [2]. Therefore, the World Health Organization (WHO) has introduced the Global Influenza Strategy for 2019–2030, which aims not only to monitor to disease prevention and control but to strengthen the low influenza vaccine uptake rates [3]. The data of influenza vaccination coverage change constantly and so present an ongoing challenge for the public health. This applies to both the general population, including higher-risk groups (e.g., the elderly, very young children, and chronically ill), and healthcare professionals (including medical students). In Poland, the ratio of seasonal influenza vaccination is one of the worst in Europe, both among the general population (approximately 4%) [4] and among members of risk groups such as the elderly (9.5%) [5], people with chronic diseases (approximately 4%) [6], medical students (from 3% to 24%) [7,8], nurses (from 5% to 10%) [9,10], and doctors (approximately 22.3%) [10]. 

In order for the WHO recommendations be implemented, therefore, it is necessary to have a positive attitude towards the flu vaccine and knowledge about the indications, contraindications, and high-risk influenza groups that get the flu among healthcare workers. This is all the more important since the flu vaccine has to be repeated annually to account for the high variability and varied types and subtypes of the flu virus [6]. The situation is not made easier by the fact that the flu vaccine in Poland, as in most other countries, is a nonobligatory and paid vaccination.

Few studies have examined HCWs’ knowledge about the flu vaccine in Poland. These studies have only presented one major (mostly medicine students) and single year of study. A survey conducted among medicine students who had clinical classes with patients revealed that a quarter of students believed the flu vaccine to be unnecessary, a fifth thought it was ineffective, and only 2% knew it was contraindications [9]. Another study found out that as much as 40% of nurses and 63% of doctors believe the flu vaccine to have a low effectiveness [10]. Although Polish students of medicine know the necessity of regular (every year) flu vaccinations, and over 80% of the respondents from this group know the CDC’s (the U.S. Centers for Disease Control and Prevention) recommendation for vaccinations of HCWs [9], according to another study, half of them did not know the contraindications against the influenza vaccination, and almost 20% feared that they would contract the flu after vaccination [11]. In addition, while 76% of the vaccinated students believed that the vaccine could reduce the number and the severity of flu infections, 11% of the group advised against taking the flu vaccination against influenza at all [11].

The effectiveness of the flu vaccine is constantly being evaluated. While vaccinations against influenza reduce the incidence of flu among children, it also protects high-risk groups and the elderly population, who may be unvaccinated [12,13,14]. Thus, the low vaccination coverage observed among medical students undergoing clinical practice, who do not want to vaccinate because they feel healthy [15] but may have an asymptomatic flu infection [16], is a serious risk for hospitalized patients [17], for the chronically ill, or for nursing home residents [18]. These students can transmit the flu virus despite being asymptomatic [19]. Therefore, it is important that HCWs and medical students are vaccinated regularly every year. The vaccination of HCWs against influenza has a positive effect on reducing respiratory tract infections in hospitals, i.e., from 32% to 3% [17], and mortality among senior citizens by 40% [18]. The effectiveness of the influenza vaccine also differs between age groups. It is much more effective among younger people (from a 70% to 90% reduction) than the elderly (30–40%) [19,20]. Therefore, the influenza vaccination should also be viewed in terms of its impact on protecting the collective interest. Knowledge of the flu vaccine among medical staff (including medical students as future HCWs) and the need to be vaccinated every year is an important factor of health promotion in society. Medical students (e.g., Nursing, Midwifery, Pharmacy, and Public health) should demonstrate good knowledge of the flu vaccine, as well as a high uptake.

The low level of flu vaccination coverage in Polish society has still remained for in recent years was the reason for a design study to find the causes of such a situation and be able to counteract them in the future. It is a large project that is covered among students from four majors and in all years of studies at a medical university. It is the first such study in Poland. In the previous paper, the rates of influenza immunization, the knowledge of students that could distinguish the flu from the common cold symptoms and the reasons for influenza vaccinations among students in these majors of study: Nursing, Midwifery, Pharmacy, and Public Health were presented [21].

The current study is the first to evaluate the level of knowledge regarding the vaccine against influenza and the attitudes towards it based on a cross-sectional survey among students in four majors of Medicine, viz., Nursing, Midwifery, Pharmacy, and Public health, at a large medical university in Poland. It also examines opinions towards the flu vaccine among vaccinated students in all years of study at the medical university.

## 2. Material and Methods

### 2.1. Participants

A cross-sectional survey was carried out over three months at the Medical University of Lodz, Poland. The self-administered survey was anonymous. All respondents were studying one of four majors: Nursing, Midwifery, Pharmacy, or Public health. Students of all years were included in the study; the Nursing, Midwifery, and Public health courses last three years, while Pharmacy lasts five years. The survey was completed by 1188 participants, but 1137 students returned fully completed questionnaires for the purposes of this study: 449 Nursing students (39.5%), 158 Midwifery students (13.8%), 442 Pharmacy students (38.9%), and 88 Public health students (7.7%). Incomplete questionnaires were discarded; a response rate of 95% was obtained.

### 2.2. Cross-Sectional Survey

A questionnaire-based cross-sectional survey was administered to determine the assessment of the vaccine against influenza among students and their understanding of the flu vaccine. This survey was conducted at the Medical University of Lodz, Poland from December 2019 to the end of February 2020. This period was chosen, because most influenza vaccinations in the country are performed from September to the end of November, and a rapid increase in the incidence of influenza is typically observed in Poland starting in December.

The participants were informed by the main investigator about the purpose of the study. Clarifying the essence of the study was important to obtain as many fully completed questionnaires as possible. The return of a blank questionnaire indicted resignation from the study. The study was designed by Sylwia Kałucka, and approval was given by the Medical University of Lodz Ethics Committee (ID: RNN/141/13/KB).

### 2.3. Questionnaire Survey

A self-administered nonstandardized questionnaire was also used. This questionnaire is a proven research tool that can be used to investigate the knowledge of influenza and influenza vaccination issues among students (healthcare workers) and/or patients in medical facilities [7,22]. Prior to use for this research, the questionnaire was improved and adapted to the present study.

The whole questionnaire consists of questions divided in several parts. The present paper only used questions about vaccinations against influenza, the effects of vaccinations, and knowledge about the flu vaccine. The first part of the research described here includes two single-answer self-assessment questions regarding the effectiveness of the flu vaccine: *Do you think that the vaccination in any way affected your health*? (yes, it had a positive effect; yes, it had a negative effect; or no, was not affected in any way) and *Did you come down with the flu or other diseases of the upper respiratory tract in the season after the vaccination*? (yes, the flu; yes, other infections; yes, both the flu and other infections; or no) and the question of whether they think it is worth getting the flu vaccine.

The second part examined their knowledge about the influenza vaccination. This was determined according to the following questions: *How often should someone be vaccinated against influenza*? (every year, every two years, every five years, or a single vaccination provides a lifelong immunity). *Does the vaccine give 100% protection against the flu*? *Does the vaccination give effective protection against upper respiratory tract infections other than influenza*? (Yes, No, or I do not know). *Could a vaccination against influenza be a direct factor causing the flu*? (yes, because the vaccine contains the influenza virus; no, because the vaccine contains inactive influenza virus; or I do not know). This part also asked which group in particular should receive the influenza vaccine: *people over 65, children under 2 years of age, chronically ill people, people living in large crowds, pregnant women,* or *frequent travelers and others* and which should not. This last question was an open-ended question, and the students were asked to enter the answers themselves.

The third part concerned the health of the respondent (i.e., chronic disease or taking medicine regularly), while the fourth part examined the participants’ demographic variables: sex, age, major (faculty) of study and year of study (among students), place of residence, and cigarette smoking status (active smoker or ex-smoker or never smoker). More details on the entirety of this survey are given elsewhere [21].

### 2.4. Statistical Analysis

Descriptive statistics were calculated using STATISTICA 13.1 software (StatSoft Inc., Krakow, Poland). Univariate and multivariate logistic regression analyses (with Wald’s chi-square test) were used to identify the predictors of attitude towards the influenza vaccine by the participants. We analyzed separate factors such as: sex (1), year of study (2), major (3), residence (4), vaccination status (5), cigarette smoking (6), status of health (7), taking medicines (8), and hospitalization (9). Statistically significant factors were those for which *p* < 0.05 in the multivariate analysis. Variables with a significance level in the univariate analysis (Tables S1–S4) were included in the multivariate analysis. Reference groups (Ref) for the categorical variables were established, for the most part of the statistical analyses, based on the lowest percentages of the “correct answer” for each category, in order for the odds ratios to be greater than one.

## 3. Results

### 3.1. The Prevalence of Flu Vaccination among Subjects and Their Demographics Characteristic

Out of 1188 questionnaires, 1137 were completed and qualified for the statistical analysis. The mean age of the participants was 21.3 ± 1.62 years. All four majors were dominated by female students (100% of Midwifery, 94.0% of Nursing, 90.9% of Public health, and 83.7% of Pharmacy). Regarding the place of residence, rural residences dominated among Nursing (35.7%), small city (below 100,000 residents) among Midwifery (34.1%) and Public Health (40.9%), and large city (above 100,000 residents) among Pharmacy (55.9%). The vast majority of the students did not smoke, and this applied to all majors (Nursing: 71.3%, Midwifery: 73.0%, Pharmacy: 81.0%, and Public Health: 62.5%). Less than a fifth of the respondents did not suffer from any chronic diseases and were not taking long-term medications. Isolated cases of hospitalization due to respiratory diseases were reported during the past 12 months: 10 cases in Nursing (2.2%), two in Midwifery (1.3%), seven in Pharmacy (1.6%), and one among Public health (1.1%) students. A detailed summary of the demographic characteristics of the subjects (gender, age, place of residence, smoking status, health status, drugs for chronic diseases, hospitalization, and vaccination status) were presented earlier [21]. Most participants were never vaccinated against the flu. The highest percentage of unvaccinated students was in Midwifery (76.0%), followed by Nursing (69.9%) and Pharmacy students (69.5%), while 51.1% of Public health students were vaccinated at least once in their lives. A detailed analysis of the flu vaccine coverage between different demographic groups was described in our previous study [21].

### 3.2. Attitude to the Influenza Vaccine among Students Who Were Vaccinated against Influenza

In our study, most students in all four majors positively assessed the flu vaccination, regardless of whether they had been vaccinated once, irregularly, or annually (Table 1). The larger group of regularly vaccinated students gave a positive assessment on the effectiveness of the vaccine: 68–89%, depending on the study major (Table 1). The positive effects of the vaccination were also reported by most of the students in Nursing (73%), Midwifery (78%), and Public health (56%) who vaccinated occasionally. Only seven of all 351 vaccinated students reported a negative effect, i.e., less than 2%.

In the season that the students were vaccinated, most did not get the flu (Table 1). Among the subjects who were vaccinated, 13 students in Nursing, 13 students in Pharmacy, three students in Midwifery, and one student in Public Health caught the flu, (2–10% of the vaccinated students).

In all the majors, the majority of the vaccinated students thought it was worth getting the flu vaccination, and this proportion grew among students who were vaccinated more regularly. Thus, 65% of the students in Pharmacy, 78% in Midwifery, and 83% in Nursing who were vaccinated once in the last three years recommended the influenza vaccination and, respectively, 85%, 90%, and 89% of students who received a regular vaccination (Table 1). Among the few students who did not think it was worth getting the flu vaccine, the prevailing opinion was that the vaccine did not prevent the disease, because the virus mutates and the vaccine may not be effective in the following season (data not shown).

### 3.3. Knowledge about the Vaccine against Influenza

The next part of the survey consisted of questions covering their basic knowledge about the flu vaccine and the effects we can expect from it. The most correct answers for the question *How often should one be vaccinated against influenza?* were given by the students in Pharmacy (83.0% vaccinated vs. 86.0% unvaccinated), followed by Midwifery (84.2% vaccinated vs. 85.0% unvaccinated) and Nursing (70.4% vaccinated vs. 74.0% unvaccinated), while the least were in Public health (44.2% vaccinated vs. 57.8% unvaccinated) (Table 2). Among the students who did not know the correct answer, the highest percentage of all the students stated that the flu vaccination should be given every two years. However, even 8.1% of the never vaccinated Pharmacy students and 20.9% Public Health students reported that vaccinations should take place every five years, and 15.5% of unvaccinated Public health students indicated that once in a lifetime was enough (Table 2).

Regardless of the vaccination status, students of Pharmacy were aware that flu vaccination do not give 100% protection against the flu (respectively, 93.3% vaccinated vs. 95.1% unvaccinated) (Table 2), followed by Midwifery (89.5% vaccinated vs. 94.2% unvaccinated) and Nursing (80.0% vaccinated vs. 85.4% unvaccinated) students. The Public health students, especially those who were vaccinated, were much less aware of this issue (58.1% vaccinated vs. 77.8% unvaccinated; *p* < 0.05) (Table 2).

Similarly, regarding the third question, *Does the vaccination give effective protection against upper respiratory tract infections other than influenza?* the worst performance was by the Public health students: the answers of only 20.9% vaccinated and 44.9% unvaccinated students were correct (*p* < 0.01). The best performance was given by Pharmacy students, although in this case, the percentage of students giving the correct answer was much lower than for the previous questions (Table 2).

The other question, regarding whether the vaccine itself can cause flu, was most often correctly answered by Pharmacy students. However, significantly more unvaccinated students of this major answered correctly (89.3%) than vaccinated students (76.3%) (*p* < 0.001) (Table 2).

On the other hand, in all majors of study, the students demonstrated poorer knowledge about the contraindications for the vaccine and the risk groups for which the vaccination is particularly recommended. At least two-thirds of Nursing and Midwifery students and more than half of Pharmacy students indicated that people over 65 years of age and people who live/work in large groups should get vaccinated (Table 3). However, far fewer Nursing, Midwifery, or Public health students indicated that people with chronic diseases should get vaccinated against influenza. Even fewer students (11.6–34%), also in Midwifery, indicated that pregnant women should receive the flu vaccine. Among the respondents who chose other, the flu vaccine was mainly recommended for medical staff.

The respondents also made a number of mistakes regarding who should not be recommended to get the flu vaccine (Table 3). Between 22% and 50% of respondents, depending on the major of study and vaccination status, did not answer the question, and between 9% to 26% indicated that no such contraindications exist. Only a few percent correctly indicated that those with acute infections or an allergy to the vaccine components are contraindications. In addition, some respondents incorrectly indicated that pregnant women, people suffering from chronic diseases, the elderly, or children should not be vaccinated.

The results of the multivariate logistic regression for individual questions regarding the students’ knowledge of the influenza vaccine are presented in Table 4, Table 5, Table 6 and Table 7. It was found that three factors appear to have a significant impact on the knowledge of the recommended frequency for the influenza vaccination: year of study, major of study, and flu vaccination status. Students in the last years of their studies were almost twice as likely to correctly answer this question than younger students (first and second years in their studies) (odds ratio (OR): 2.19, 95% confidence interval (CI) [1.47–3.27], *p* < 0.001) (Table 4). Public health students had the poorest knowledge. Midwifery and Pharmacy students were five times more likely to answer this question correctly than Public health students (respectively, OR: 5.43, 95% CI [2.91–10.13], *p* < 0.001 and OR: 5.16, 95% CI [3.10–8.60], *p* < 0.001), and Nursing students were almost four times more likely (OR: 3.76, 95% CI [2.29–6.16], *p* < 0.001). Students who were never vaccinated against influenza were almost 1.5 times more likely to answer correctly than those who were (OR: 1.38, 95% CI [1.00–1.90], *p* < 0.001) (Table 4).

Correct responses that the flu vaccine does not give 100% protection against influenza were connected with four factors: year of study, major of study, place of residence, and vaccination status (Table 5). Additionally, in this case, obviously, students in higher years were more likely to answer correctly than those in lower ones; second-year students gave the correct answer twice as frequently as the first-year students (OR: 2.28, 95% CI [1.44–3.60], *p* < 0.001), while the final-year students (third–fifth) were almost three times more likely (OR: 2.89, 95% CI [1.76–4.74], *p* < 0.001). Compared to Public health students, Pharmacy students were nearly eight times more likely to be correct (OR: 7.64, 95% CI [4.00–14.59], *p* < 0.001), Midwifery students more than six times (OR: 6.19, 95% CI [2.81–13.64], *p* < 0.001), and Nursing students nearly three times (OR: 2.66, 95% CI [1.54–4.61], *p* < 0.001). In addition, students living in small cities (OR: 2.25, 95% CI [1.40–3.62], *p* < 0.001) and large cities (OR: 2.24, 95% CI [1.42–3.54], *p* < 0.001) were twice as likely to give correct answers than students living in rural areas (Table 5).

The results of the univariate and multivariate logistic regressions regarding their knowledge of significance of the flu vaccine in relation to other upper respiratory tract infections other than influenza are given in Table 6. Again, the students of Nursing and Midwifery were almost twice as likely to answer correctly than those of Public Health (OR: 1.88, 95% CI [1.16–3.05], *p* = 0.011 and OR: 1.84, 95% CI [1.07–3.18], *p* = 0.029), and those of Pharmacy were nearly four times as likely (OR: 3.82, 95% CI [2.32–6.29], *p* < 0.001).

The only significant factor influencing students’ knowledge about the influenza vaccine as a potential causative agent of the disease was the major of study (Table 7). Pharmacy students were nearly five times more likely to choose the correct answer (OR: 4.87, 95% CI [2.93–8.10], *p* < 0.001), Midwifery students four times (OR: 4.04, 95% CI [2.23–7.31], *p* < 0.001), and Nursing students more than twice as likely (OR: 2.24, 95% CI [1.39–3.61], *p* < 0.001) than Public Health students (Table 7).

## 4. Discussion

The current study evaluated their knowledge of the flu vaccine and attitude towards it among vaccinated medical students. Unlike previous research in Poland, the present work examined four majors of study (Nursing, Midwifery, Pharmacy, and Public health) and all years of study.

Medical students need knowledge of the flu vaccine to effectively promote influenza vaccination (e.g., frequency vaccinate, efficacy, and protection), especially since this vaccine is paid and nonmandatory. It is not enough to know that the vaccination reduces morbidity due to influenza 10-fold [23] if every third-vaccinated Public health student (34.9%) believes it is enough to be vaccinated every two years and 15.5% believe that a single vaccination provides lifelong immunity. Therefore, the low vaccination coverage observed among students of all four majors every year is not surprising. However, in contrast to Public health students, most vaccinated and unvaccinated students of Pharmacy, Midwifery, and Nursing recognize the importance of annual flu vaccinations. On the other hand, all respondents demonstrated relatively poor knowledge regarding the strong recommendations and contraindications of the influenza vaccine. The differences observed in their knowledge about the influenza vaccine among students in these four majors of study, and particularly the lower level of knowledge among Public health students, was the reason why the programs of study analysis was performed. The programs of study in Midwifery and Nursing include, among others, Microbiology, Epidemiology, Primary care, and Public health. They provide a basic knowledge of bacteriology and virology and the social aspects of vaccination. The programs of study in Pharmacy include additional Immunology, which discusses in detail the immunoprophylaxis against infectious diseases. The program of studies in Public health include, among other subjects: Epidemiology, which primarily concerns the methods used in epidemiological research and the knowledge of population health data. The subjects such as Infectious diseases and their global threats mainly include the issues of the National Sanitary Inspection structures (e.g., isolation procedures, quarantine, etc.), and only a short time is spent on the etiology, microbiology, and vaccination issues regarding infectious diseases. Therefore, the partial misunderstanding of the subject of the flu vaccine in Public health students may be due to a poorer basic knowledge of Microbiology and Immunology. Meanwhile, it is the Public health students who are currently being prepared for health promotion, including primary prevention by vaccination [24].

Healthcare workers, including students of Pharmacy, Nursing, Midwifery, and Public health, are responsible for providing correct knowledge about the influenza vaccine in society to maintain a high quality of medical services in healthcare institutions and to protect patients against vaccine-preventable diseases. Therefore, it is worrying that some participants believe that flu vaccination can itself cause flu infection. Similar results have been obtained in previous studies [24,25].

Our results demonstrated that only a few students in all four majors receive seasonal flu vaccinations each year. The total rate of vaccination coverage amongst the students was also very low, below one-third in Pharmacy, Nursing, and Midwifery, the same values as in other countries: Hong Kong, Spain, and Italy [26,27,28], and below 50% in Public health. This low uptake of the flu vaccine among students has previously been associated with them having a strong sense of their own good health [15,24]. They do not feel the need to take the flu vaccine, since they do not often get sick [29]. In the described study, a few to a dozen percent of the surveyed students of Nursing, Midwifery, and Pharmacy even considered young age and good health as contraindications to the vaccination (Table 3).

Moreover, this is disturbing, because medical students do not have enough awareness that they may act as vectors of the flu infection towards their patients [30]. Similarly, HCWs in the UK have a low level of concern around influenza and a low perception of the benefits of influenza vaccinations [31]. They may often have influenza asymptomatically [16], making it even more difficult to convince them to vaccinate. In particularly, this concerns nurses and midwives who having major caregiving roles among patients, and in our study, they showed the lowest vaccination coverage. Moreover, a study in the UK showed that HCWs perceive influenza to be a much greater risk to their patients than for themselves [31]. Raising awareness among students that they themselves are also at a high risk of catching the flu, and with the vaccination, they can protect not only themselves but other vulnerable groups in society during clinical practice has been found to have a positive effect on their willingness to get vaccinated [15,32].

A combination of education about the responsibility for oneself and others, and an awareness of the need for annual flu vaccinations, can have a positive effect. A study in Hong Kong showed that the chances of getting vaccinated were over 17 times higher for those who had at least one flu vaccine than for those who were not vaccinated at all [26]. Another study in Singapore found educational activities regarding the flu vaccine increased the proportion of vaccinated healthcare professionals from 56.8% to 66.4% [33]. Some research has found that, among healthcare professionals, the influenza vaccination is perceived as less important than other vaccinations, such as for tuberculosis (TB) and hepatitis B (HBV) [34]. However, vaccination of the individual or medical staff against influenza is associated with a social responsibility to protect others, especially the seriously ill and people in hospitals [35].

Our present findings indicate that Pharmacy, Midwifery, and Nursing students were more likely to be aware that the flu vaccination did not give 100% protection than the Public health students, who again demonstrated less knowledge of the flu vaccine. Despite this, the participants had a positive attitude towards the flu vaccine. It is also important to ensure that as many influenza vaccinations as possible are performed on medical students every year. Previous research has shown that students who vaccinate annually or who have vaccinated more than once in the past showed more willingness to vaccinate the next season [36]. In addition, almost all vaccinated students in the present study, regardless of whether they vaccinated once or regularly, gave a positive assessment of the vaccination; however, the flu vaccine and its positive effects were rated better by students who were vaccinated regularly.

The annual flu epidemic requires regular annual vaccinations. Health habits such as regular flu vaccinations and hand hygiene [37,38] need to be presented constantly to be better accepted by students and the rest of society during influenza pandemics [39,40]. In Brazil, an awareness-raising activity of the flu vaccine increased the percentage of vaccinated medical staff to 34%; however, the lack of its continuation caused a decrease to 20% in the next year and to 12% after two years [41]. HCWs (including medical students) need to promote positive behaviors, starting with themselves. These positive habits should develop when they are still students and should be continued during their clinical practice and future medical careers [36]. During the course of their studies, students should acquire complete knowledge of vaccinology in order to understand that hand washing is not more important than the flu vaccine [42]. It found that education can increase vaccination coverage by up to four times [42].

Our findings indicate that, in all majors, knowledge about the flu vaccine grows with the year of study, with the senior students having a significantly better understanding than the youngest students. Similar findings have been confirmed in other studies (e.g., the same among medical students in the USA). However, we revealed that the higher levels of knowledge about the vaccine among students of Nursing, Pharmacy, and Midwifery do not guarantee the same high vaccine uptakes in these majors [24]. On the contrary, a significantly higher rate of vaccination was observed in the younger years of study.

To conclude, our findings highlight three key points: the participants demonstrated a positive assessment of the flu vaccine effectiveness and good knowledge of the flu vaccine but poor flu vaccination coverage. Our analysis provided a completely new picture of knowledge about the primary prevention of the flu vaccine by medical students. It seems important to focus on younger healthcare workers, with low influenza uptakes, to benefit all of society. Therefore, we recommend that students should receive during each year of study instruction and training at the end of their study about primary prevention as the best method of preventing serious influenza complications in order to develop pro-health attitudes in future HCWs.

## Figures and Tables

**Table 1:** Self-assessment of vaccine effectiveness among vaccinated students from four majors: Nursing, Midwifery, Pharmacy, and Public health.

Vaccinated	Major							
	Nursing N = 135		Midwifery N = 38		Pharmacy N = 135		Public Health N = 43	
	Once N = 90	Regularly * N = 45	Once N = 18	Regularly * N = 20	Once N = 55	Regularly * N = 80	Once N = 9	Regularly * N = 34
Vaccination effect								
Yes, it had a positive effect	66 (73.3%)	40 (88.9%)	14 (77.8%)	14 (70%)	24 (43.6%)	58 (72.5%)	5 (55.6%)	23 (67.6%)
Yes, it had a negative effect	2 (2.2%)	0 (0.0%)	0 (0.0%)	2 (10%)	2 (3.6%)	0 (0.0%)	1 (11.1%)	0 (0.0%)
No, it did not affect my health in any way	22 (24.5%)	5 (11.1%)	4 (22.2%)	4 (20%)	29 (52.7%)	22 (27.5%)	3 (33.3%)	11 (33.4%)
Morbidity after vaccination								
Yes, the flu	7 (7.8%)	4 (8.9%)	1 (5.6%)	1 (5%)	3 (5.4%)	5 (6.3%)	1 (11.1%)	0 (0.0%)
Yes, the other infections	20 (22.2%)	7 (17.8%)	2 (11.1%)	5 (25%)	8 (14.6%)	28 (35%)	0 (0.0%)	10 (29.4%)
Yes, both flu and other infections	2 (2.2%)	0 (0.0%)	0 (0.0%)	1 (5.0%)	1 (1.8%)	4 (5%)	0 (0.0%)	0 (0.0%)
No	61 (67.8%)	33 (73.3%)	15 (83.3%)	13 (65%)	43 (78.2%)	43 (53.7%)	8 (88.9%)	24 (70.6%)
Vaccinate against flu								
No	6 (6.7%)	0 (0.0%)	2 (11.1%)	2 (10%)	11 (20%)	7 (8.7%)	0 (0.0%)	1 (2.9%)
Yes	75 (83.3)	40 (88.9%)	14 (77.8%)	18 (90%)	36 (65.5%)	67 (83.8%)	9 (100%)	31 (91.2%)
No answer	9 (10%)	5 (11.1%)	2 (11.1%)	0 (0.0%)	8 (14.5%)	6 (7.5%)	0 (0.0%)	2 (5.9%)

* At least twice within the last 3 years.

**Table 2:** Knowledge of the vaccination among vaccinated and unvaccinated students from four majors: Nursing, Midwifery, Pharmacy, and Public health.

Influenza Vaccination	Major							
	Nursing N = 449		Midwifery N = 158		Pharmacy N = 442		Public Health N = 88	
	Vaccinated	Unvaccinated	Vaccinated	Unvaccinated	Vaccinated	Unvaccinated	Vaccinated	Unvaccinated
	135 (30.1%)	314 (69.9%)	38 (24.0%)	120 (76%)	135 (30.5%)	307 (69.5%)	43 (48.9%)	45 (51.1%)
How often should you be vaccinated against influenza?								
**every year**	95 ** (70.4%)	258 (82.2%)	32 (84.2%)	102 (85.0%)	112 (83.0%)	264 (86.0%)	19 (44.2%)	26 (57.8%)
every two years	22 * (16.3%)	28 (8.9%)	3 (7.9%)	10 (8.3%)	10 (7.4%)	30 (9.8%)	15 (34.9%)	9 (20.0%)
every five years	10 (7.4%)	17 (5.4%)	0 (0.0%)	4 (3.3%)	11 * (8.1%)	11 (3.6%)	9 (20.9%)	3 (6.7%)
every ten years	5 (3.7%)	4 (1.3%)	3 (7.9%)	2 (1.7%)	0 (0.0%)	2 (0.6%)	0 (0.0%)	0 (0.0%)
single vaccination provides lifelong immunity	3 (2.2%)	7 (2.2%)	0 (0.0%)	2 (1.7%)	2 (1.5%)	0 (0.0%)	0* (0.0%)	7 (15.5%)
Does the vaccination give 100% protection against the flu?								
Yes	13 (9.6%)	16 (5.1%)	1 (2.6%)	2 (1.7%)	2 (1.5%)	6 (2.0%)	9 (20.9%)	5 (11.1%)
**No**	108 (80.0%)	268 (85.4%)	34 (89.5%)	113 (94.2%)	126 (93.3%)	292 (95.1%)	25 * (58.1%)	35 (77.8%)
I do not know	14 (10.4%)	30 (9.6%)	3 (7.9%)	5 (4.2%)	7 (5.2%)	9 (2.9%)	9 (20.9%)	5 (11.1%)
Does the vaccination give effective protection against upper respiratory tract infection other than influenza?								
Yes	20 (14.8%)	42 (13.4%)	7 (18.4%)	14 (11.7%)	15 (11.1%)	29 (9.5%)	13 (30.2%)	8 (17.8%)
**No**	65 (48.2%)	165 (52.5%)	24 (63.2%)	57 (47.5%)	89 (65.9%)	226 (73.6%)	9 ** (20.9%)	22 (48.9%)
I do not know	50 (30.7%)	107 (34.1%)	7 * (18.4%)	49 (40.8%)	31 (23.0%)	52 (16.9%)	21 (48.9%)	15 (33.3%)
Could vaccination against influenza be a direct factor causing the flu?								
Yes	15 (11.1%)	27 (8.6%)	3 (7.9%)	5 (4.2%)	15 (11.1%)	24 (7.8%)	4 (9.3%)	5 (11.1%)
**No**	99 (73.3%)	222 (70.7%)	29 (76.3%)	101 (84.2%)	103 *** (76.3%)	274 (89.3%)	22 (51.2%)	23 (51.1%)
I do not know	21 (15.6%)	65 (20.7%)	6 (15.8%)	14 (11.7%)	17 *** (12.6%)	9 (2.9%)	17 (39.5%)	17 (37.8%)

* *p* < 0.05, ** *p* < 0.01, and *** *p* < 0.001 vaccinated vs. unvaccinated; correct answers in **bold**.

**Table 3:** Recommendations and contraindications for influenza vaccination among vaccinated and unvaccinated students from four majors: Nursing, Midwifery, Pharmacy, and Public health.

	Major							
	Nursing N = 449		Midwifery N = 158		Pharmacy N = 442		Public Health N = 88	
	Vaccinated	Unvaccinated	Vaccinated	Unvaccinated	Vaccinated	Unvaccinated	Vaccinated	Unvaccinated
	135 (30.1%)	314 (69.9%)	38 (24.0%)	120 (76%)	135 (30.5%)	307 (69.5%)	43 (48.9%)	45 (51.1%)
Who should get vaccinated:								
elderly people over 65 years of age	84 *** (62.2%)	258 (82.2%)	27 (71.1%)	91 (75.8%)	74 *** (54.8%)	235 (76.5%)	18 (41.9%)	26 (57.8%)
children under 2 years of age	73 (54.1%)	155 (49.4%)	21 * (55.3%)	41 (34.2%)	83 *** (61.5%)	127 (41.4%)	28 (65.1%)	25 (55.6%)
patients with chronic diseases	57 * (42.2%)	177 (56.4%)	15 (39.5%)	61 (50.8%)	75 *** (55.6%)	223 (72.6%)	15 (34.9%)	11 (24.4%)
working in large clusters of people	91 (67.4%)	201 (64.0%)	31 (81.6%)	84 (70.0%)	82 (60.7%)	182 (59.3)	22 (51.2%)	18 (40.0%)
women in the 2nd and 3rd trimesters of pregnancy	33 * (24.4%)	106 (33.8%)	7 (18.4%)	27 (22.5%)	25 (18.5%)	81 (26.4%)	5 (11.6%)	11 (24.4%)
frequent travelers	49 (36.3%)	87 (27.7%)	15 * (39.5%)	28 (23.3%)	42 (31.1%)	101 (32.9%)	16 (37.2%)	15 (33.3%)
Other	3 * (2.2%)	27 (8.6%)	4 (10.5%)	10 (8.3%)	0 ** (0.0%)	23 (7.5%)	0 (0.0%)	2 (4.4%)
Who should not get vaccinated:								
patients with active infection	19 (14.1%)	50 (15.9%)	3 (7.9%)	18 (15%)	11 (8.1%)	30 (9.8%)	5 (11.6%)	0 (0.0%)
allergic to the components of the vaccine	15 (11.1%)	28 (8.9%)	2 (5.2%)	1 (0.8%)	23 *** (17%)	21 (6.8%)	1 (2.3%)	2 (4.4%)
patients with reduced immunity	11 (8.1%)	33 (10.5%)	8 (21%)	17 (14.2%)	24 (17.8%)	62 (20.2%)	7 (16.3%)	8 (17.8%)
everyone should	31 (23%)	62 (19.7%)	6 (15.8%)	13 (10.8%)	18 * (13.3%)	67 (21.8%)	11 (25.6%)	4 (8.9%)
pregnant women	4 (3%)	22 (7%)	2 (5.2%)	11 (9.2%)	11 (8.1%)	15 (4.9%)	4 (11.4%)	4 (8.9%)
young and healthy people	5 (3.7%)	19 (6.1%)	1 (2.6%)	17 14.2%)	10 (7.4%)	12 (3.9%)	0 (0.0%)	0 (0.0%)
children under 2 years of age	4 (3%)	11 (3.5%)	4 (10.5%)	8 (6.7%)	2 (1.5%)	3 (1%)	0 (0.0%)	4 (8.9%)
other	15 (11.1%)	20 (6.3%)	9 * (23.7%)	11 (9.2%)	2 (1.5%)	7 (2.3%)	5 (11.6%)	8 (17.8%)
No answer	29 (21.5%)	79 (25.2%)	19 (50%)	40 (33.3%)	63 ** (46.7%)	99 (32.2%)	13 (30.2%)	17 (37.8%)

* *p* < 0.05, ** *p* < 0.01, and *** *p* < 0.001 vaccinated vs. unvaccinated.

**Table 4:** Associations between the demographic factors (sex, major, year of study, place of residence, smoking cigarettes, status health, taking medication, and vaccination status) and student knowledge about the flu vaccination frequency.

	Total	Correct Answer	Multivariate Logistic Regression	
			OR (95% CI)	*p*
Sex			Factor nonstatistically significant in univariate logistic regression	
Female	1032	828 (80.2%)		
Male	105	80 (76.2%)		
Major				
Nursing	449	353 (78.6%)	3.76 (2.29–6.16)	<0.001
Midwifery	158	134 (84.8%)	5.43 (2.91–10.13)	<0.001
Pharmacy	442	376 (85.1%)	5.16 (3.10–8.60)	<0.001
Public health	88	45 (51.1%)	1.0 Ref	
Year of study				
1st	371	278 (74.9%)	1.0 Ref	
2nd	350	264 (75.4%)	1.00 (0.70–1.42)	0.990
3rd + 4th + 5th	416	366 (88.0%)	2.19 (1.47–3.27)	<0.001
Place of residence			Factor nonstatistically significant in univariate logistic regression	
rural	358	286 (79.9%)		
city to 100,000 r	305	246 (80.7%)		
city above 100,000 r	474	376 (79.3%)		
Cigarette smoking			Factor nonstatistically significant in univariate logistic regression	
current or ex-smoker	287	234 (81.5%)		
never smoker	850	674 (79.3%)		
Status health–chronic disease				
No, any	945	745 (78.8%)	1.0 Ref	
Yes	192	163 (84.9%)	1.13 (0.62–2.03)	0.693
Taking medication for chronic disease				
Yes	206	179 (86.9%)	1.68 (0.93–3.06)	0.088
No	931	729 (78.3%)	1.0 Ref	
Hospitalization due to respiratory diseases			Factor nonstatistically significant in univariate logistic regression	
Yes	20	16 (80.0%)		
No	1117	892 (79.9%)		
Vaccination status				
Unvaccinated	786	650 (82.7%)	1.38 (1.00–1.90)	<0.001
Vaccinated	351	258 (73.5%)	1.0 Ref	

The multivariate model included variables at a significance level of *p* < 0.05 in the univariate analysis (Table S1). r—resident, OR—odds ratio, and CI—confidence interval.

**Table 5:** Associations between the demographic factors (sex, major, year of study, place of residence, smoking cigarettes, status health, taking medication, and vaccination status) and the students’ knowledge about the effectiveness of the influenza vaccination.

	Total	Correct Answer	Multivariate Logistic Regression	
			OR (95% CI)	*p*
Sex			Factor nonstatistically significant in univariate logistic regression	
Female	1032	907 (87.9%)		
Male	105	94 (89.5%)		
Major				
Nursing	449	376 (83.7%)	2.66 (1.54–4.61)	<0.001
Midwifery	158	147 (93.0%)	6.19 (2.81–13.64)	<0.001
Pharmacy	442	418 (94.6%)	7.64 (4.00–14.59)	<0.001
Public Health	88	60 (68.2%)	1.0 Ref	
Year of study				
1st	371	296 (79.8%)	1.0 Ref	
2nd	350	316 (90.3%)	2.28 (1.44–3.60)	<0.001
3rd + 4th + 5th	416	389 (93.5%)	2.89 (1.76–4.74)	<0.001
Place of residence				
rural	358	292 (81.6%)	1.0 Ref	
city to 100,000 r	305	271 (88.9%)	2.25 (1.40–3.62)	<0.001
city above 100,000 r	474	438 (92.4%)	2.24 (1.42–3.54)	<0.001
Cigarette smoking			Factor nonstatistically significant in univariate logistic regression	
current or ex-smoker	287	246 (85.7%)		
never smoker	850	755 (88.8%)		
Status health–chronic disease			Factor non-statistically significant in univariate logistic regression	
No, any	945	833 (88.2%)		
Yes	192	168 (87.5%)		
Taking medication for chronic disease			Factor nonstatistically significant in univariate logistic regression	
Yes	206	185 (89.8%)		
No	931	816 (87.7%)		
Hospitalization due to respiratory diseases			Factor nonstatistically significant in univariate logistic regression	
Yes	20	20 (100%)		
No	1117	981 (87.8%)		
Vaccination status				
Unvaccinated	786	708 (90.1%)	1.47 (0.99–2.20)	0.058
Vaccinated	351	293 (83.5%)	1.0 Ref	

The multivariate model included variables at a significance level of *p* < 0.05 in the univariate analysis (Table S2). r—resident, OR—odds ratio, and CI—confidence interval.

**Table 6:** Associations between the demographic factors (sex, major, year of study, place of residence, smoking cigarettes, status health, taking medication, and vaccination status) and the students’ knowledge about the effectiveness of the flu vaccine in protecting against other upper respiratory tract infections.

	Total	Correct Answer	Multivariate Logistic Regression	
			OR (95% CI)	*p*
Sex				
Female	1032	588 (57.0%)	1.0 Ref	
Male	105	69 (65.7%)	1.17 (0.74–1.84)	0.492
Major				
Nursing	449	230 (51.2%)	1.88 (1.16–3.05)	0.011
Midwifery	158	81 (51.3%)	1.84 (1.07–3.18)	0.029
Pharmacy	442	315 (71.3%)	3.82 (2.32–6.29)	<0.001
Public Health	88	31 (35.2%)	1.0 Ref	
Year of study				
1st	371	204 (55.0%)	1.0 Ref	
2nd	350	180 (51.4%)	0.84 (0.62–1.14)	0.255
3rd + 4th + 5th	416	273 (65.6%)	1.29 (0.95–1.75)	0.106
Place of residence				
rural	358	199 (55.6%)	1.0 Ref	
city to 100,000 r	305	155 (50.8%)	0.92 (0.67–1.27)	0.620
city above 100,000 r	474	303 (63.9%)	1.22 (0.91–1.64)	0.189
Cigarette smoking				
current or ex-smoker	287	150 (52.3%)	1.0 Ref	
never smoker	850	507 (59.7%)	1.15 (0.87–1.53)	0.331
Status health–chronic disease			Factor nonstatistically significant in univariate logistic regression	
No, any	945	549 (58.1%)		
Yes	192	108 (56.3%)		
Taking medication for chronic disease			Factor nonstatistically significant in univariate logistic regression	
Yes	206	128 (62.1%)		
No	931	529 (56.8%)		
Hospitalization due to respiratory diseases			Factor nonstatistically significant in univariate logistic regression	
Yes	20	11 (55.0%)		
No	1117	646 (57.8%)		
Vaccination status				
Unvaccinated	786	470 (59.8%)	1.22 (0.93–1.60)	0.156
Vaccinated	351	187 (53.3%)	1.0 Ref	

The multivariate model included variables at a significance level of *p* < 0.05 in the univariate analysis (Table S3). r—residents, OR—odds ratio, and CI—confidence interval.

**Table 7:** Associations between the demographic factors (sex, major, year of study, place of residence, smoking cigarettes, status health, taking medication, and vaccination status) and the students’ knowledge about the possibility of influenza being caused by the flu vaccine.

	Total	Correct Answer	Multivariate Logistic Regression	
			OR (95% CI)	*p*
Sex			Factor nonstatistically significant in univariate logistic regression	
Female	1032	787 (76.3%)		
Male	105	86 (81.9%)		
Major				
Nursing	449	321 (71.5%)	2.24 (1.39–3.61)	<0.001
Midwifery	158	130 (82.3%)	4.04 (2.23–7.31)	<0.001
Pharmacy	442	377 (85.3%)	4.87 (2.93–8.10)	<0.001
Public Health	88	45 (51.1%)	1.0 Ref	
Year of study				
1st	371	270 (72.8%)	1.0 Ref	
2nd	350	277 (79.1%)	1.35 (0.94–1.92)	0.104
3rd + 4th + 5th	416	326 (78.4%)	1.06 (0.75–1.51)	0.730
Place of residence				
rural	358	267 (74.6%)	1.0 Ref	
city to 100,000 r	305	217 (71.2%)	0.93 (0.65–1.33)	0.690
city above 100,000 r	474	389 (82.1%)	1.36 (0.96–1.93)	0.081
Cigarette smoking			Factor nonstatistically significant in univariate logistic regression	
current or ex-smoker	287	218 (76.0%)		
never smoker	850	655 (77.1%)		
Status health–chronic disease			Factor nonstatistically significant in univariate logistic regression	
No, any	945	728 (77.0%)		
Yes	192	145 (75.5%)		
Taking medication for chronic disease			Factor nonstatistically significant in univariate logistic regression	
Yes	206	157 (76.2%)		
No	931	716 (76.9%)		
Hospitalization due to respiratory diseases			Factor nonstatistically significant in univariate logistic regression	
Yes	20	16 (80.0%)		
No	1117	857 (76.7%)		
Vaccination status				
Unvaccinated	786	620 (78.8%)	1.33 (0.98–1.82)	0.068
Vaccinated	351	253 (72.1%)	1.0 Ref	

The multivariate model included variables at a significance level of *p* < 0.05 in the univariate analysis (Table S4). r—residents, OR—odds ratio, and CI—confidence interval.

## Supplementary Materials

The following are available online at https://www.mdpi.com/1660-4601/18/4/2105/s1, Table S1: Associations between demographic factors (sex, major, year of study, place of residence, smoking cigarettes, status health, taking medication, and vaccination status) and student knowledge about flu vaccination frequency—univariate logistic regression. Table S2: Associations between demographic factors (sex, major, year of study, place of residence, smoking cigarettes, status health, taking medication, vaccination status) and student knowledge about the effectiveness of influenza vaccination—univariate logistic regression. Table S3: Associations between demographic factors (sex, major, year of study, place of residence, smoking cigarette, status health, taking medication, vaccination status) and student knowledge about the effectiveness of the flu vaccine in protecting against other respiratory tract infections—univariate logistic regression. Table S4: Associations between demographic factors (sex, major, year of study, place of residence, smoking cigarette, status health, taking medication, vaccination status) and student knowledge about the possibility of influenza being caused by the flu vaccine- univariate logistic regression.
