# Supplementary material for: Knowledge, Beliefs and Attitudes towards the Influenza Vaccine among Future Healthcare Workers in Poland
Source: Int J Environ Res Public Health. 2021 Feb 22;18(4):2105. doi: 10.3390/ijerph18042105 (PMC7926487; doi:10.3390/ijerph18042105)
Supplement: Supplementary file 1 [file ijerph-18-02105-s001.pdf]

Table S1. Associations between demographic factors (sex, major, year of study, place of residence, smoking cigarette, status health, taking medication, vaccination status) and student knowledge about flu vaccination frequency—univariate logistic regression.

	Total	Correct Answer	Univariate Logistic Regression	
			OR 95% CI	*p*
**Sex**				
Female	1032	828 (80.2%)	1.29 (0.79–2.04)	0.326
Male	105	80 (76.2%)	1.0 Ref.	
**Major**				
Nursing	449	353 (78.6%)	3.51 (2.18–5.65)	<0.001
Midwifery	158	134 (84.8%)	5.34 (2.92–9.76)	<0.001
Pharmacy	442	376 (85.1%)	5.44 (3.32–8.92)	<0.001
Public health	88	45 (51.1%)	1.0 Ref	
**Year of study**				
1st	371	278 (74.9%)	1.0 Ref	
2nd	350	264 (75.4%)	1.03 (0.73–1.44)	0.878
3rd+4th+5th	416	366 (88.0%)	2.45 (1.68–3.57)	<0.001
**Place of residence**				
rural	358	286 (79.9%)	1.04 (0.74–1.46)	0.841
city to 100,000 r	305	246 (80.7%)	1.09 (0.76–1.56)	0.651
city above 100,000 r	474	376 (79.3%)	1.0 Ref	
**Cigarette smoking**				
current or ex–smoker	287	234 (81.5%)	1.15 (0.82–1.62)	0.417
never smoker	850	674 (79.3%)	1.0 Ref	
**Status health –chronic disease**				
No, any	945	745 (78.8%)	1.0 Ref	
Yes	192	163 (84.9%)	1.51 (0.99–2.31)	0.058
**Taking medication for chronic disease**				
Yes	206	179 (86.9%)	1.84 (1.19–2.84)	0.006
No	931	729 (78.3%)	1.0 Ref	
**Hospitalization due to respiratory diseases**				
Yes	20	16 (80.0%)	1.01 (0.33–3.05)	0.987
No	1117	892 (79.9%)	1.0 Ref	
**Vaccination status**				
Unvaccinated	786	650 (82.7%)	1.72 (1.28–2.33)	<0.001
Vaccinated	351	258 (73.5%)	1.0 Ref	

r—resident

Table S2. Associations between demographic factors (sex, major, year of study, place of residence, smoking cigarette, status health, taking medication, vaccination status) and student knowledge about the effectiveness of influenza vaccination—univariate logistic regression.

	Total	Correct Answer	Univariate Logistic Regression	
			**OR 95% CI**	***p***
**Sex**				
Female	1032	907 (87.9%)	1.0 Ref	
Male	105	94 (89.5%)	1.18 (0.61–2.26)	0.623
**Major**				
Nursing	449	376 (83.7%)	2.40 (1.44–4.02)	0.001
Midwifery	158	147 (93.0%)	6.24 (2.91–13.35)	<0.001
Pharmacy	442	418 (94.6%)	8.13 (4.41–14.96)	<0.001
Public Health	88	60 (68.2%)	1.0 Ref	
**Year of study**				
1st	371	296 (79.8%)	1.0 Ref	
2nd	350	316 (90.3%)	2.35 (1.52–3.64)	<0.001
3rd+4th+5th	416	389 (93.5%)	3.65 (2.29–5.82)	<0.001
**Place of residence**				
rural	358	292 (81.6%)	1.0 Ref	
city to 100,000 r	305	271 (88.9%)	1.80 (1.15–2.81)	<0.001
city above 100,000 r	474	438 (92.4%)	2.75 (1.78–4.24)	<0.001
**Cigarette smoking**				
current or ex–smoker	287	246 (85.7%)	1.0 Ref	
never smoker	850	755 (88.8%)	1.32 (0.89–1.96)	0.162
**Status health –chronic disease**				
No, any	945	833 (88.2%)	1.06 (0.66–1.70)	0.801
Yes	192	168 (87.5%)	1.0 Ref	
**Taking medication for chronic disease**				
Yes	206	185 (89.8%)	1.24 (0.76–2.03)	0.389
No	931	816 (87.7%)	1.0 Ref	
**Hospitalization due to respiratory diseases**				
Yes	20	20 (100%)	–	–
No	1117	981 (87.8%)	–	–
Vaccination status				
Unvaccinated	786	708 (90.1%)	1.80 (1.25–2.59)	0.002
Vaccinated	351	293 (83.5%)	1.0 Ref	

r—resident

Table S3. Associations between demographic factors (sex, major, year of study, place of residence, smoking cigarette, status health, taking medication, vaccination status) and student knowledge about the effectiveness of the flu vaccine in protecting against other upper respiratory tract infections—univariate logistic regression.

	Total	Correct Answer	Univariate Logistic Regression	
			OR 95% CI	*p*
**Sex**				
Female	1032	588 (57,0%)	1.0 Ref	
Male	105	69 (65,7%)	1.45 (0.95–2.21)	0.086
**Major**				
Nursing	449	230 (51.2%)	1.93 (1.20–3.11)	0.007
Midwifery	158	81 (51.3%)	1.93 (1.13–3.31)	0.016
Pharmacy	442	315 (71.3%)	4.56 (2.81–7.40)	<0.001
Public Health	88	31 (35.2%)	1.0 Ref	
**Year of study**				
1st	371	204 (55.0%)	1.0 Ref	
2nd	350	180 (51.4%)	0.87 (0.65–1.16)	0.333
3rd+4th+5th	416	273 (65.6%)	1.56 (1.17–2.08)	0.002
**Place of residence**				
rural	358	199 (55.6%)	1.0 Ref	
city to 100,000 r	305	155 (50.8%)	0.83 (0.61–1.12)	0.220
city above 100,000 r	474	303 (63.9%)	1.42 (1.07–1.87)	0.015
**Cigarette smoking**				
current or ex–smoker	287	150 (52.3%)	1.0 Ref	
never smoker	850	507 (59.7%)	1.35 (1.03–1.77)	0.029
**Status health–chronic disease**				
No, any	945	549 (58.1%)	1.08 (0.79–1.48)	0.637
Yes	192	108 (56.3%)	1.0 Ref	
**Taking medication for chronic disease**				
Yes	206	128 (62.1%)	1.24 (0.91–1.70)	0.163
No	931	529 (56.8%)	1.0 Ref	
**Hospitalization due to respiratory diseases**				
Yes	20	11 (55.0%)	1.0 Ref	
No	1117	646 (57.8%)	1.12 (0.46–2.73)	0.799
**Vaccination status**				
Unvaccinated	786	470 (59.8%)	1.30 (1.01–1.68)	0.040
Vaccinated	351	187 (53.3%)	1.0 Ref	

r—resident

Table S4. Associations between demographic factors (sex, major, year of study, place of residence, smoking cigarette, status health, taking medication, vaccination status) and student knowledge about the possibility of influenza being caused by the flu vaccine—univariate logistic regression.

	Total	Correct Answer	Univariate Logistic Regression	
			OR 95% CI	*p*
**Sex**				
Female	1032	787 (76.3%)	1.0 Ref	
Male	105	86 (81.9%)	1.41 (0.84–2.36)	0.194
**Major**				
Nursing	449	321 (71.5%)	2.40 (1.50–3.82)	<0.001
Midwifery	158	130 (82.3%)	4.44 (2.47–7.97)	<0.001
Pharmacy	442	377 (85.3%)	5.54 (3.38–9.09)	<0.001
Public Health	88	45 (51.1%)	1.0 Ref	
**Year of study**				
1st	371	270 (72.8%)	1.0 Ref	
2nd	350	277 (79.1%)	1.42 (1.01–2.00)	0.047
3rd+4th+5th	416	326 (78.4%)	1.35 (0.98–1.88)	0.069
**Place of residence**				
rural	358	267 (74.6%)	1.0 Ref	
city to 100,000 r	305	217 (71.2%)	0.84 (0.60–1.19)	0.321
city above 100,000 r	474	389 (82.1%)	1.56 (1.12–2.18)	0.009
**Cigarette smoking**				
current or ex–smoker	287	218 (76.0%)	1.0 Ref	
never smoker	850	655 (77.1%)	1.06 (0.78–1.46)	0.702
**Status health –chronic disease**				
No, any	945	728 (77.0%)	1.09 (0.76–1.56)	0.650
Yes	192	145 (75.5%)	1.0 Ref	
**Taking medication for chronic disease**				
Yes	206	157 (76.2%)	1.0 Ref	
No	931	716 (76.9%)	1.04 (0.73–1.48)	0.831
**Hospitalization due to respiratory diseases**				
Yes	20	16 (80.0%)	1.21 (0.40–3.67)	0.731
No	1117	857 (76.7%)	1.0 Ref	
**Vaccination status**				
Unvaccinated	786	620 (78.8%)	1.45 (1.08–1.93)	0.013
Vaccinated	351	253 (72.1%)	1.0 Ref	

r—residents
